# Polymorphism of angiogenesis regulation factor genes (VEGF/VEGFR), and extracellular matrix remodeling genes (MMP/TIMP), and the levels of their products in extracellular tissues of patients with primary and secondary lymphedema

**DOI:** 10.18699/vjgb-24-49

**Published:** 2024-07

**Authors:** V.I. Konenkov, V.V. Nimaev, A.V. Shevchenko, V.F. Prokofiev

**Affiliations:** Research Institute of Clinical and Experimental Lymрhology – Branch of the Institute of Cytology and Genetics, Siberian Branch of the Russian Academy of Sciences, Novosibirsk, Russia; Research Institute of Clinical and Experimental Lymрhology – Branch of the Institute of Cytology and Genetics, Siberian Branch of the Russian Academy of Sciences, Novosibirsk, Russia; Research Institute of Clinical and Experimental Lymрhology – Branch of the Institute of Cytology and Genetics, Siberian Branch of the Russian Academy of Sciences, Novosibirsk, Russia; Research Institute of Clinical and Experimental Lymрhology – Branch of the Institute of Cytology and Genetics, Siberian Branch of the Russian Academy of Sciences, Novosibirsk, Russia

**Keywords:** primary lymphedema, secondary lymphedema, VEGF, MMP, TIMP, KDR, NRP, лимфедема первичная, лимфедема вторичная, VEGF, MMP, TIMP, KDR, NRP

## Abstract

Cells of various organs and systems perform their functions and intercellular interactions not in an inert environment, but in the microenvironment of tissue fluids. Violations of the normal drainage of tissue fluids accompany lymphedema. An important mechanism of angiogenesis and vasculogenesis regulation in tissue fluids is the production and reception of vascular endothelial growth factors in combination with the regulation of matrix metalloproteinases. The aim of the work was to perform: a comparative analysis of some polymorphisms of vascular endothelial growth factor and their receptors and the genes encoding matrix metalloproteinases in two forms of lymphedema; an analysis of the relationship of these genes’ polymorphisms with the levels of vascular endothelial growth factor and matrix metalloproteinases and their inhibitors in serum and affected tissues. Polymorphism of VEGF (rs699947, rs3025039), KDR (rs10020464, rs11133360), NRP2 (rs849530, rs849563, rs16837641), matrix metalloproteinases MMP2 (rs2438650), MMP3 (rs3025058), MMP9 (rs3918242), Timp1 (rs6609533) and their combinations were analyzed by the Restriction Fragment Length Polymorphism method and TaqMan RT-PCR. The serum and tissue fluid levels were determined using the ELISA test system. Changes in the frequency distribution of MMP2 genotypes in primary and MMP3 in secondary lymphedema are shown. Significant frequency differences in NRP2 genotypes were revealed by comparing primary and secondary lymphedema. Features of the distribution of complex genotypes in primary and secondary lymphedema were revealed. The correlation analysis revealed the interdependence of the concentrations of the MMP, TIMP and VEGF products and differences in the structure of the correlation matrices of patients with both forms of lymphedema. It was shown that, in primary lymphedema, genotypes associated with low MMP2 and TIMP2 in serum and tissue fluid are detected, while in secondary lymphedema, other associations of the production levels with combined genetic traits are observed.

## Abstract (ru)

Клетки различных органов и систем осуществляют свои функции и межклеточные взаимодействия не в инертной среде, а в микроокружении тканевых жидкостей. Нарушения нормального дренажа тканевых жидкостей сопровождают лимфедему. Важный механизм регуляции ангиогенеза и васкулогенеза в подкожной клетчатке – выработка и рецепция факторов роста эндотелия сосудов в сочетании с регуляцией матриксных металлопротеиназ. Цель настоящего исследования: сравнительный анализ полиморфизма генов фактора роста эндотелия сосудов и его рецепторов вместе с генами матриксных металлопротеиназ при двух формах лимфедемы, анализ взаимосвязи полиморфизма этих генов с уровнем фактора роста эндотелия сосудов и матриксных металлопротеиназ и их ингибиторов в сыворотке крови и пораженных тканях. Полиморфизм VEGF (rs699947, rs3025039), рецепторов к нему KDR (rs10020464, rs11133360), NRP2 (rs849530, rs849563, rs16837641), матриксных металлопротеиназ MMP2 (rs2438650), MMP3 (rs3025058), MMP9 (rs3918242), ингибитора металлопротеиназ Timp1 (rs6609533) и их комбинаций проанализирован методами анализа длин рестрикционных фрагментов и TaqMan RT-PCR. Уровень белков в сыворотке и тканевой жидкости определяли с использованием тест-систем ELISA. Показаны изменения частот распределения генотипов ММР2 при первичной и ММР3 при вторичной лимфедеме. Высокодостоверные различия частот генотипов NRP2 обнаружены при сравнении первичной и вторичной лимфедемы. Выявлены особенности распределения «комплексных» генотипов при первичной и вторичной лимфедеме. Корреляционный анализ показал взаимозависимость концентрации исследуемых белковых продуктов MMP, TIMP и VEGF и выраженные различия в структуре корреляционных матриц пациентов с обеими формами лимфедемы. Продемонстрировано, что при первичной лимфедеме выявляются генотипы, ассоциированные с низкими значениями MMP2 и TIMP2 в сыворотке крови и тканевой жидкости, а при вторичной лимфедеме – иные связи концентраций исследуемых белков с комбинированными генетическими признаками.

## Introduction

In recent years, the interest of researchers in the state of the extracellular matrix and the vascular bed of the circulatory and lymphatic systems immersed in it has been constantly growing. The number of scientific publications has been increasing more than 5–6 times a year over the past 40 years. This is due to the understanding that cells of various organs and systems, with their complex internal metabolism, carry out the most important functions and intercellular interactions not in an inert environment, but in a constant microenvironment of tissue fluids carrying a huge number of regulatory factors of the most diverse secreted and membrane-associated nature.

Violations of the normal drainage of tissue fluids lead to tissue hypoxia and a variety of edematous syndromes accompanying various pathological changes, ranging from inflammation to tumor growth. A striking example of impaired drainage of tissue fluid is lymphedema (Miller, 2020). It is represented by both a predominantly genetically determined “primary” and a “secondary” form associated with post-mastectomy consequences or chronic venous insufficiency (Poveshchenko et al., 2010; Quirion, 2010). According to some estimates, between 140 and 200 million people worldwide suffer from lymphedema (Forte et al., 2019). Despite such a wide spread of this disease and numerous studies in this area, the main treatment method is comprehensive physical decongestant therapy and lifelong supportive use of compression knitwear (Vignes, 2017; Executive Committee…, 2020). One of the factors of such, relatively speaking, pathogenetic therapy is, in our opinion, the lack of clear ideas about vascular disorders in the functioning of the blood and lymphatic channels and their interaction with the extracellular matrix, leading to obstruction of the physiological outflow of tissue fluid in the affected regions.

The most important mechanism for the regulation of angiogenesis and vasculogenesis in subcutaneous tissue is the production and reception of the VEGF vascular endothelial growth factor system, represented by VEGF-A, VEGF-B, VEGF-C, VEGF-D, PGF and the VEGFR-1 (Flt-1), VEGFR- 2 (Flk-1/KDR), VEGFR-3 (Flt-4) receptor families to them (Vaahtomeri et al., 2017; Rauniyar et al., 2018). Their interaction ensures the growth, remodeling and functioning of the circulatory and lymphatic systems. The genes of these proteins are polymorphic, which affects the level of their expression, affinity and functional activity (Luo et al., 2019; Yap et al., 2019).

It has been established that the VEGF-A ligand binds and transmits signals through the VEGFR-1 and VEGFR-2 receptors, whereas VEGF-B transmits signals exclusively through VEGFR-1, and VEGF-C and VEGF-D have high affinity for VEGFR-3. In addition, there are two neuropilin receptors, which are transmembrane glycoproteins that function on the VEGF-VEGFR axis. Neuropilin-1 (NRP-1), a non-kinase coreceptor for VEGFR-2, functions to enhance the binding and signaling of certain isoforms of VEGF-A, and NRP-2 is a non-kinase coreceptor for VEGFR-3 (Mucka et al., 2016; Stevens, Oltean, 2019; Gao Y. et al., 2020). Understanding the genetic mechanisms underlying endothelial apoptosis and lymphangiogenesis will shed light on the role of disruption of these processes in the development of chronic inflammation and transformation of connective tissue in lymphedema (Saik et al., 2019).

The family of matrix metalloproteinases (MMP) is directly related to the processes of angiogenesis and the activity of regulatory growth factors of the vascular endothelium. The activity of these tissue enzymes is controlled by their tissue inhibitor system (TIMP) (Cabral-Pacheco et al., 2020). The genes encoding them also are widely polymorphic, and protein products are expressed on lymphatic endothelial cells and degrade the collagen of the vascular endothelial lining (Detry et al., 2012). Previously, numerous data were obtained on the effect of regulatory factor gene polymorphism on cell expression and production (Watson et al., 2000; Gao X. et al., 2019).

The aim of this study is a comparative analysis of polymorphism of genes of vascular endothelial growth factor and its receptors together with genes of matrix metalloproteinases in two forms of lymphedema, analysis of the relationship of polymorphism of these genes with the level of vascular endothelial growth factor and matrix metalloproteinases and their inhibitors in blood serum and affected tissues.

## Materials and methods

Patients. The study included patients with the confirmed diagnosis of lymphedema of the extremities. The recruitment of patients was carried out on the basis of Research Institute of Clinical and Experimental Lymрhology – Branch of the Institute of Cytology and Genetics, Siberian Branch of the Russian Academy of Sciences (RICEL) in the period from January 2017 to November 2018. The sample of 174 patients (18–81 years old, median age 52 years) was divided into primary (72 people) and secondary limb lymphedema (102 people). The division of groups into primary and secondary lymphedema is based on etiological signs according to clinical recommendations (Executive Committee…, 2020).

The criteria for primary lymphedema of the extremities were considered to be the development of clinical manifestations without connection with such etiological factors as removal of lymph nodes, radiation therapy, trauma or surgical intervention in the projection of lymphatic collectors. The appearance of clinically significant edema due to a single episode of erysipelas, which is considered a provoking factor against the background of insufficiency of the functional reserve of the lymphatic region. The sample of patients with secondary lymphedema included patients with lesions of both upper and lower extremities. The majority of patients with secondary lymphedema of the upper extremities underwent complex treatment of breast cancer (66 patients – 97.1 %), in 2 patients the cause of edema of the upper limb was the combined treatment of lymphosarcoma of all groups of peripheral lymph nodes and mediastinum IIIA st, lymphogranulomatosis with lesions of the cervical and axillary lymph nodes. The appearance of edema after repeated recurrence of erysipelas was attributed to secondary post-inflammatory lymphedema.

All patients included in the study had no progression or recurrence of a malignant tumor, and belonged to the 3rd clinical group of dispensary observation. According to the classification of the International Society of Lymphologists, stage 2 of the disease prevailed. The third stage of the disease was represented by 7 % of the primary lymphedema group and 8.9 % of the secondary lymphedema group. Informed consent to participate in the study was signed by each participant of the study. The protocol of the clinical research was approved by the RICEL Local Ethics Committee (Primary Protocol No. 127 dated 01/13/2017). Blood serum and interstitial fluid were collected in the morning, on an empty stomach, from patients admitted to the RICEL clinic for a course of complex decongestant therapy, before it began. Patients with current or recent erysipelas were not included in the study. The clinical characteristics of the patients are presented in Table 1.

**Table 1.**
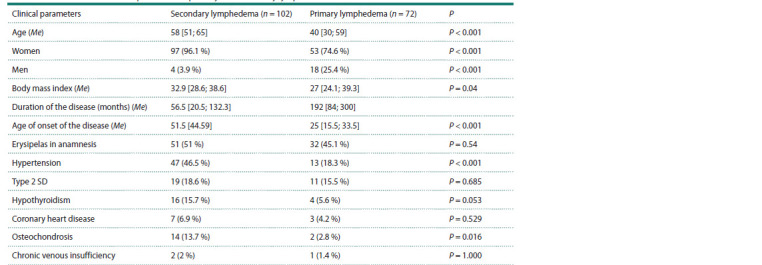
Clinical characteristics of patients with primary and secondary lymphedema Notе. Me is the median; the interquartile range is indicated in square brackets; percentages are indicated in parentheses.

The comparison group consisted of 339 people of comparable gender and age, residents of the Novosibirsk region. Relatives were not included in either the patient groups or the comparison group.

Genotyping. Genotyping of VEGF-2578 (rs699947) and MMP9-1562 (rs3918242) was performed by the Restriction Fragment Length Polymorphism (RFLP) method. The structure of the primers, restriction endonucleases and the product size are shown in Table 2.

**Table 2.**
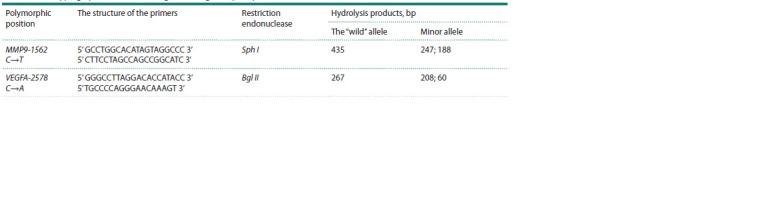
Genotyping by the Restriction Fragment Length Polymorphism method

Gene polymorphism of VEGF+936 (rs3025039), NRP2 13581 (rs849530), NRP2 68279 (rs849563), NRP2 92646 (rs16837641), KDR 17693 (rs10020464), KDR 14011 (rs11133360), MMP2-1306 (rs2438650), MMP3-1171 (rs3025058), Timp1 (rs rs6609533) was analyzed by Real- Time PCR using commercial test systems of TaqMan probe method (Syntol, Russia) on the DT-96 thermocycler (DNA Technology, Russia) according to the instructions

Enzyme-linked immunosorbent assay (ELISA). Quantitative determination of vascular endothelial growth factor, metalloproteinases and their inhibitors was carried out by ELISA kits (ng/mL) according to the instructions: Human VEGF (Quantikine ELISA, R&D Systems, USA) MMP-3 (AESKU Diagnostics, Germany), TIMP-3 (Brand Owner CLOUD-CLONE CORP., USA), MMP-2 and TIMP-2 (Quantikine ELISA, R&D Systems, USA).

Statistical analysis. Statistical processing was carried out using specialized IBM SPSS Statistics 23 (USA) and the software package for volumetric processing of bioinformatics, including multidimensional genetic analysis. In the statistical analysis of the results of the genetic study, the frequency of occurrence of alleles, genotypes and their polylocus combinations, the odds ratio (OR), and the 95 % confidence interval for OR (OR’s 95 % CI) were calculated.

The distribution of genotypes was checked with the Hardy– Weinberg equilibrium. The significance level of differences in the frequency in the compared groups was determined by the two-sided criterion of the exact Fisher method for fourfield tables, with Bonferroni correction. Considering that the distribution of most of the studied quantitative features was different from normal, nonparametric statistical methods were used. The intergroup differences were assessed using the Mann–Whitney U-test and the Kraskel–Wallis ANOVA. Intra-group differences were assessed using the Wilcoxon sign rank criterion for related samples. Spearman’s rank correlation method was used to analyze the strength and direction of the correlation between pairs of features.

The description of quantitative variables is presented in the form of median (Me) and interquartile range (the interval between the 25th [Q 0.25] and 75th [Q 0.75] percentiles). The hypothesis of the normal distribution of quantitative parameters was tested using the Shapiro–Wilk criterion and the Kolmogorov–Smirnov criterion with the Lilliefors correction. The mathematical processing of the relationship of genetic traits with quantitative laboratory parameters was carried out in accordance with the methodological approaches of quantile analysis. With this approach, ranges above p75 (upper quartile, Q3) and lower ranges below p25 (lower quartile, Q1) were taken as parameters of an increased concentration of indicators. The critical level of significance when testing statistical hypotheses was assumed to be 0.05.

## Results

The analysis of the distribution of the analyzed complex genetic traits among patients with primary lymphedema revealed a number of pronounced differences from the conditionally “normal” distribution established by us in the study of a significant group of healthy individuals without signs of lymphatic edema of the extremities. When analyzing the degree of differences between individual variants of the studied genetic parameters, they were established only for MMP2-1306, associated with C predominance among the patient ( p = 0.029). Along with this, when analyzing the frequency of occurrence of combined genetic traits, including polymorphic variants of both the MMP and VEGF genes, significantly more pronounced differences were revealed when comparing groups of patients with primary lymphedema and healthy individuals (Table 3).

**Table 3.**
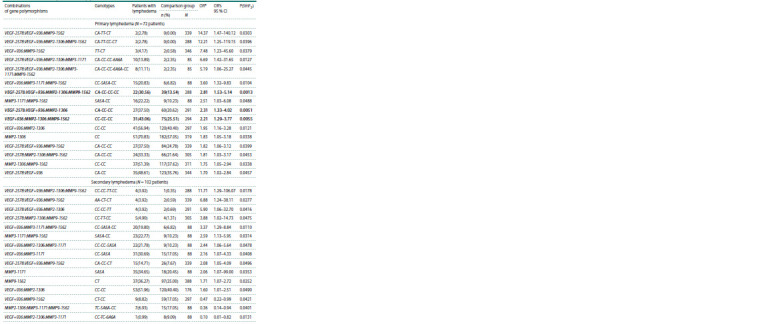
Frequency of distribution of individual and complex MMP and VEGF genotypes among patients with primary and secondary lymphedema Notе. The comparison group for primary and secondary lymphedema N = 339, OR – odds ratio; OR’s 95 % CI – 95 % confidence interval for OR; P(tmF2) – level of statistical significance (p) of differences according to the exact Fisher test (two–sided). * The data in the table are sorted in descending OR, significant differences p < 0.01 are highlighted in bold.

Basically, these differences were associated with an increase in almost all the analyzed signs in the patient with a level of reliability of differences in the range from 0.048 to 0.001 according to the two-sided Fisher exact test. At the same time, such combinations of genotypes as VEGF-2578 CA: VEGF+936 TT: MMP9-1562 CT and VEGF-2578 CA: VEGF+936 TT: MMP2-1306 CC: MMP9-1562 CT were not found among representatives of the group of healthy individuals and were identified exclusively among patients with primary lymphedema. With a certain degree of probability, they can be attributed to the “genetic markers” of an individual’s constitutional predisposition to the development of primary lymphedema. Further research is needed to test this hypothesis.

When conducting a similar analysis with secondary lymphedema, in the development of which anatomical factors that occur during surgical damage to the lymphatic and circulatory pathways of the outflow of tissue fluid are of greater importance, we get a different picture from the previous group. In these patients, associative links between the development of the disease and variants of the MMP2 gene are no longer found, but deviations from the “normal” distribution of the MMP3 and MMP9 genes in the analyzed sites are revealed. Thus, the predominance of MMP3-1171 5A5A and MMP9- 1562 CT genotypes was revealed. The frequency of the combination of these homozygous genotypes is also twice as high among these patients relative to the control group.

The inclusion of VEGF in the analysis increases the degree of differences between the patients and the controls. Thus, the frequency of a combined genetic trait represented by a combination of homozygous VEGF+936 CC: MMP3-1171 5A5A: MMP9-1562 CC among the patients with secondary lymphedema exceeds the frequency of a similar indicator in the control group by more than 3 times (OR = 3.37; p = 0.0110). An even more “broad” combined genetic trait, including a combination of homozygous VEGF-2578 CC: VEGF+936 CC: MMP2-1306 TT: MMP9-1562 CC, is more often detected among patients with secondary lymphedema (OR = 11.71; p = 0.0178). Along with this, the frequency of another genetic trait represented by a combination of VEGF+936 CC: MMP2- 1306 TC: MMP3-1171 6A6A in the patient group is almost 10 times lower: from 9.09 % in the control group to 0.99 % in the patient group (OR = 0.10; p = 0.0131). The presence of this combination in the human genome can to some extent be considered a protective factor.

In order to analyze the differences between the structural parameters of angiogenesis genes in more detail, we conducted a study of the distribution of combined signs in both groups of patients with primary and secondary lymphedema. In this report, we have included data on polymorphisms of the KDR genes in two polymorphic positions, the NRP gene in three polymorphic positions and the TIMPI gene. For a clearer representation of the data on the differences obtained, the data with p < 0.005 are presented (Table 4).

**Table 4.**
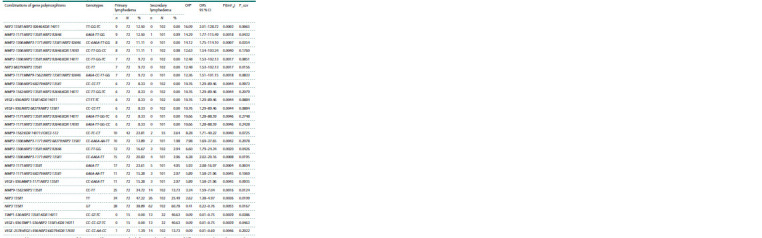
Comparative analysis of the distribution of individual and complex genotypes between groups with primary and secondary lymphedema Notе. OR – odds ratio; OR’s 95 % CI – 95 % confidence interval for OR; P(tmF2) – level of statistical significance (p) of differences according to the exact Fisher test (two–sided); P_cor – adjusted value of P(tmF2) (taking into account the Bonferroni correction). * The data in the table are sorted in descending order of the OR value

When evaluating the results, attention is drawn to the presence of combined genetic signs in both groups of patients, which alternatively are not detected in the compared samples. Thus, in the group with primary lymphedema, TIMP1-536 CC: NRP2 13581 GT: KDR 14011 TC and VEGF+936 CC: TIMP1-536 CC: NRP2 13581 GT: KDR 14011 TC are completely absent. In both cases, these combinations are quite widely represented (more than 40 %) in the group with secondary lymphedema ( p = 0.0039). In contrast to these data, in the group of patients with secondary lymphedema, there are completely no combined genetic signs represented by combinations NRP2 13581 TT: NRP2 92646 GG: KDR 14011 TC; MMP2-1306 CC: MMP3-1171 6A6A: NRP2 13581 TT: NRP2 92646 GG; MMP2-1306CC: NRP2 13581 TT: NRP2 92646 GG: KDR 14011 TC; MMP3-1171 6A6A: MMP9-1562 CC: NRP2 13581 TT: NRP2 92646 GG and a number of others. The significance level of the differences is 0.0003–0.005.

Taking into account the previously obtained numerous data on the effect of regulatory factor gene polymorphism on expression and production, we conducted a study of the MMP 1, 2, 3, 9 proteins level; their tissue inhibitors TIMP 1, 2, 3 level and VEGF level, which did not reveal significant differences between groups of patients with primary and secondary lymphedema according to the median bilateral Mann–Whitney U criterion. At the same time, a continuous correlation analysis revealed not only the interdependence of the analyzed protein MMP, TIMP and VEGF levels, but also pronounced differences in the structure of the correlation matrices of patients with both forms of lymphedema.

Thus, in primary lymphedema, the most significant correlations are revealed between MMP2 and the tissue inhibitor TIMP2 levels (OR = 0.703; p < 0.01), whereas the VEGF serum level is inversely correlated with the MMP3 serum level (OR = –0.629; p < 0.05). In secondary lymphedema, the most significant interdependencies are revealed between the MMP2 and TIMP2 extracellular fluid levels (OR = 0.727; p < 0.01). The VEGF serum level is inversely correlated with this growth factor and MMP9 extracellular fluid level (data are not presented in the table). Other direct and inverse correlations between the signs are also revealed, which probably indicates the functioning of the unified system of humoral factors involved in the processes of angiogenesis and lymphangiogenesis.

Taking into account the presented data on a pronounced associative relationship between the analyzed complex genetic traits and various forms of lymphedema, we conducted an additional analysis of the dependence of high or low MMP 1, 2, 3, 9 of their tissue inhibitors TIMP 1, 2, 3 and VEGF proteins levels on the presence of various combined genotypes in patients of both groups (Table 5).

**Table 5.**
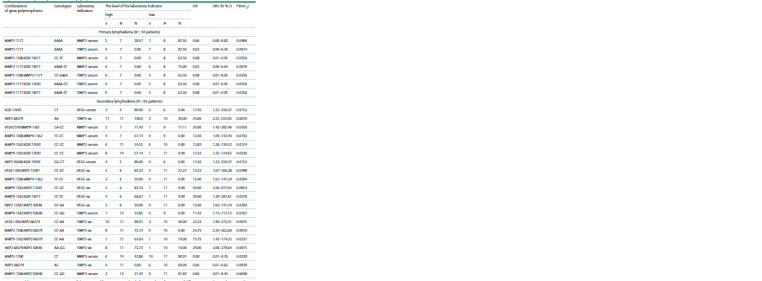
Relationship between gene polymorphisms and laboratory parameters levels in serum and tissues of patients with primary and secondary lymphedema Notе. OR – odds ratio; OR’s 95 % CI – 95 % confidence interval for OR; P(tmF2) – level of statistical significance (p) of differences according to the exact Fisher test (two–sided); ex – extracellular fluid.

The conducted quantile analysis showed that in primary lymphedema, genotypes associated exclusively with low MMP2 and TIMP2 level are detected both in the serum and in the extracellular fluid of patients. In the group of patients with secondary lymphedema, other multidirectional associations of the proteins levels with combined genetic traits are shown, which are absent in the group of patients with primary lymphedema.

## Discussion

Primary lymphedema occurs as a result of an isolated or developing congenital anomaly of the lymphatic system as part of the syndrome and is associated with dysplasia, hypoplasia or hyperplasia of components of the lymphatic system. The lower extremities are affected in primary lymphedema in the vast majority of cases (Gordon et al., 2021). Secondary lymphedema develops as a complication of another disease or intervention as a result of a violation of the anatomical integrity or obliteration of lymphatic collectors, removal or lesion of lymph nodes, followed by impaired lymph outflow, lymph stasis in lymph vessels and increased endolymphatic pressure (Executive Committee…, 2020). Therefore, it is important to understand the genetic characteristics of these disorders.

In primary lymphedema, we found an increase in the frequency of the MMP2-1306C allelic variant of the gene and the homozygous CC variant in a single genotype and in other 10 out of 17 combined genetic traits, the frequency of which is higher in this form of the disease with an obvious genetic predisposition (Poveshchenko et al., 2010; Shevchenko et al., 2020).

MMP2 is one of the zinc-dependent endopeptidases that were first discovered as proteases targeting and cleaving extracellular proteins. However, the intracellular significance of MMP has also been discussed over the past 20 years, and research on a new aspect of their functions has been expanding (Bassiouni et al., 2021). Polymorphism of the MMP2-1306 gene plays a significant role in carcinogenesis, in particular, the C variant is associated with a protective role in the development of prostate cancer (Zhang et al., 2017), its frequency is higher among patients with bronchial asthma (Chen et al., 2020). There are a number of reports on changes in the frequency of variants of this polymorphic gene in the promoter region and with other diseases; however, we present data on its association with the development of primary lymphedema for the first time.

Also, for the first time, we present data on changes in the frequency of the MMP3-1171 5A/6A gene in lymphedema. Thus, among patients with secondary lymphedema, 5A5A in the composition of combined signs is characteristic, the frequency of which is higher in this form of the disease, along with 6A6A in the composition of signs, the frequency of which is lower in secondary lymphedema. There is no such pattern in primary lymphedema. The discriminating role of the homozygous MMP3-1171 6A6A is clearly manifested in the analysis of data comparing the distribution of combined genetic traits between groups of patients with primary and secondary forms of lymphedema. We believe that the identified phenomenon requires further study and more detailed clinical analysis

According to the data presented in Table 3, MMP3-1171 6A6A, both as a single trait and as part of a number of combined genetic traits, is associated with low MMP2 and TIMP2 serum levels in patients with primary lymphedema. The homozygous MMP2-1306 CC in the composition of combined genetic traits is also associated with low MMP2 and TIMP2 serum levels in primary lymphedema

In patients with secondary lymphedema, MMP2 C is already associated with a high MMP1 and MMP3 serum level, with high VEGF and TIMP3 levels in extracellular fluid. The dependence of any level of the analyzed quantitative signs with the MMP3-1171 gene polymorphism in secondary lymphedema, unlike its primary form, was not established.

It can be concluded that genetic factors associated with the family of VEGF genes and their VEGFR receptors involved in the regulation and development of vascular networks of the lymphatic and circulatory systems play a significant role in the development of primary lymphedema.

The VEGFR3 receptor performs the main function in the development and formation of the lymphatic system. Autosomal dominant mutations of VEGFR3, which interfere with the functioning of the receptor as a homodimer, not only cause one of the main forms of hereditary primary lymphostasis, namely primary lymphedema (Milroy’s disease), but also participate in predisposition to the development of common cyanotic congenital heart defects, demonstrating a new function of VEGFR3 in the early development of heart tissues (Monaghan et al., 2021). This explains the interest in studying the role of a number of VEGFR family genes (especially NRP-2) associated with the development of angiogenesis and vasculogenesis, endothelial growth factor genes VEGF, metalloproteinases MMP and their tissue inhibitors TIMP.

As a result of research, there is increasing evidence that in the development of secondary lymphedema, which develops as a result of surgical vascular disorders such as mastectomy, genetic factors of predisposition to the development of lymphatic edema of the extremities also play a role, which allows us to hope for the creation of prognostic criteria for identifying groups at increased risk of their development and preventive measures. The practical significance and prospects of such studies are the positive results of the developed therapy of lymphedema, including with the help of inducers of lymphangiogenesis with VEGF drugs (Forte et al., 2019).

## Conclusion

Among patients with both primary and secondary lymphedema, there are significant deviations from the normative indicators established for the control group of healthy individuals in the frequency of distribution of a number of complex genotypes of the MMP 2, 3, 9 and VEGF genes, which indicates a significant influence of the studied fragment of the patient’s genotype on predisposition to these types of lymphatic edematous syndrome.

The groups of patients with primary and secondary lymphedema differ significantly in the nature of the distribution of a number of complex genotypes of the MMP 2, 3, 9 and VEGF genes, which indicates numerous ways of realizing a genetic predisposition to the development of these pathological conditions

Comparative analysis revealed no significant differences in the level of matrix metalloproteinases, their tissue inhibitors and vascular endothelial growth factor in serum and extracellular fluid of patients with primary and secondary lymphedema.

In both primary and secondary lymphedema, various associative relationships have been established between the studied combined genotypes of gene polymorphism of angiogenesis regulation factors and the level of protein products of these genes in serum and extracellular fluid, which in turn indicates the presence of certain genomic and metabolomic mechanisms for the realization of a genetic predisposition to the development of lymphatic edema.

Data on an increase in the frequency of homozygous MMP2-1306 CC in primary lymphedema and an increase in the frequency of homozygous MMP3-1171 5A5A in secondary lymphedema were obtained. Both of these polymorphisms are associated with quantitative indicators of the content of protein products MMP, TIMP and VEGF in various variants of limb lymphedema development.

## Conflict of interest

The authors declare no conflict of interest.
